# Supplementary material for: Amyloid Burden Correlates with Electrocardiographic Findings in Patients with Cardiac Amyloidosis—Insights from Histology and Cardiac Magnetic Resonance Imaging
Source: J Clin Med. 2024 Jan 9;13(2):368. doi: 10.3390/jcm13020368 (PMC10816127; doi:10.3390/jcm13020368)
Supplement: Supplementary file 1 [file jcm-13-00368-s001.zip › jcm-2801783-supplementary.pdf]

Table S1. Cox regression analyses for the composite endpoint of all-cause death, heart transplantation, cardiovascular-related hospitalization.

**Variable**	**Crude hazard ratio**	**95% Confidence interval**	***P* value**
	**Univariable regression**		
**Clinical and laboratory parameters**			
Body mass index, kg/m^2 (IQR)	1.049	0.989 – 1.114	0.114
New York Heart Association class ≥III, n (%)	1.456	0.914 – 2.321	0.114
6-Minute walk distance, m (IQR)	0.998	0.996 – 1.000	0.067
**Log N-terminal pro brain natriuretic peptide, pg/mL (IQR)**	1.456	1.186 – 1.787	**≤0.001**
Troponin T, ng/L	1.011	1.005 – 1.017	**≤0.001**
Estimated glomerular filtration rate, mL/min/1.73m2 (IQR)	0.996	0.986 – 1.006	0.421
National Amyloidosis Center stage I-III	1.297	0.952 – 1.768	0.099
**Electrocardiographical parameters**			
Total lead voltage, mm (IQR)	0.995	0.987 – 1.004	0.265
Low QRS voltage	1.270	0.768 – 2.102	0.352
Heart rate, bpm (IQR)	1.009	0.994 – 1.025	0.251
PQ interval, ms (IQR)	1.008	0.999 – 1.017	0.086
QRS width, ms (IQR)	1.000	0.992 – 1.008	0.980
QT time, ms (IQR)	1.001	0.994 – 1.008	0.737
AV-Block, n (%)	1.056	0.642 – 1.737	0.831
Any bundle branch block, n (%)	0.901	0.569 – 1.425	0.655
Incomplete left bundle branch block, n (%)	0.669	0.265 – 1.692	0.396
Complete left bundle branch block, n (%)	0.653	0.348 – 1.225	0.184
Complete right bundle branch block, n (%)	1.930	0.764 – 4.879	0.165
**Left anterior fascicular block, n (%)**	1.131	0.489 – 2.618	0.773
Bifascicular block n (%)	1.546	0.667 – 3.585	0.309

Anterior pseudoinfarct pattern, n (%)	0.966	0.530 – 1.761	0.911
Ventricular premature complexes, n (%)	1.360	0.621 – 2.979	0.442
**Cardiac magnetic resonance imaging parameters**			
**Extracellular volume, % (IQR)**	1.020	1.003 – 1.037	**0.024**
LVEDD, mm (IQR)	1.039	1.000 – 1.079	0.053
**RVEDD, mm (IQR)**	1.037	1.002 – 1.073	**0.037**
Interventricular septum, mm (IQR)	1.022	0.969 – 1.078	0.419
Left atrial area, cm^2 (IQR)	1.004	0.981 – 1.027	0.749
**Right atrial area, cm^2 (IQR)**	1.045	1.014 – 1.077	**0.004**
Ascending aorta, mm (IQR)	1.010	0.957 – 1.066	0.715
Pulmonary artery, mm (IQR)	1.050	0.997 – 1.106	0.065
Left ventricular ejection fraction, % (IQR)	0.983	0.965 – 1.002	0.080
Left ventricular end-diastolic volume, mL (IQR)	1.000	0.995 – 1.005	0.990
Left ventricular cardiac output, L/min (IQR)	1.003	0.835 – 1.206	0.972
Left ventricular mass, g (IQR)	1.002	0.998 – 1.007	0.349
**Right ventricular ejection fraction, % (IQR)**	0.972	0.953 – 0.992	**0.006**
**Right ventricular end-diastolic volume, mL (IQR)**	1.006	1.001 – 1.011	**0.018**
Right ventricular cardiac output, L/min (IQR)	1.112	0.946 – 1.308	0.197

IQR indicates interquartile range.
